# Transcriptome and functional analysis revealed the intervention of brassinosteroid in regulation of cold induced early flowering in tobacco

**DOI:** 10.3389/fpls.2023.1136884

**Published:** 2023-03-31

**Authors:** Xiumei Dai, Yan Zhang, Xiaohong Xu, Mao Ran, Jiankui Zhang, Kexuan Deng, Guangxin Ji, Lizeng Xiao, Xue Zhou

**Affiliations:** ^1^ College of Agronomy and Biotechnology, Southwest University, Chongqing, China; ^2^ Engineering Research Center of South Upland Agriculture, Ministry of Education, Chongqing, China; ^3^ Chongqing Tobacco Science Research Institute, Chongqing, China

**Keywords:** tobacco, cold, early flowering, brassinosteroid, *NtBRI1*

## Abstract

Cold environmental conditions may often lead to the early flowering of plants, and the mechanism by cold-induced flowering remains poorly understood. Microscopy analysis in this study demonstrated that cold conditioning led to early flower bud differentiation in two tobacco strains and an Agilent Tobacco Gene Expression microarray was adapted for transcriptomic analysis on the stem tips of cold treated tobacco to gain insight into the molecular process underlying flowering in tobacco. The transcriptomic analysis showed that cold treatment of two flue-cured tobacco varieties (Xingyan 1 and YunYan 85) yielded 4176 and 5773 genes that were differentially expressed, respectively, with 2623 being commonly detected. Functional distribution revealed that the differentially expressed genes (DEGs) were mainly enriched in protein metabolism, RNA, stress, transport, and secondary metabolism. Genes involved in secondary metabolism, cell wall, and redox were nearly all up-regulated in response to the cold conditioning. Further analysis demonstrated that the central genes related to brassinosteroid biosynthetic pathway, circadian system, and flowering pathway were significantly enhanced in the cold treated tobacco. Phytochemical measurement and qRT-PCR revealed an increased accumulation of brassinolide and a decreased expression of the flowering locus c gene. Furthermore, we found that overexpression of *NtBRI1* could induce early flowering in tobacco under normal condition. And low-temperature-induced early flowering in *NtBRI1* overexpression plants were similar to that of normal condition. Consistently, low-temperature-induced early flowering is partially suppressed in *NtBRI1* mutant. Together, the results suggest that cold could induce early flowering of tobacco by activating brassinosteroid signaling.

## Introduction

Flowers develop from florally determined meristems that proliferate to form the floral organs which include the sepals, petals, stamen, and carpels. The importance of transitional time from vegetative growth to flowering is paramount because flowering is the first step of sexual reproduction (Bernier et al., 1993). Numerous genes have been identified that can change the basic pattern of flower morphogenesis, indicating that flower differentiation is under strict genetic control. Transcription factors related to development that regulate specific downstream gene targets may trigger a response in plants which promotes initiation of flowering. Flower differentiation appears to be controlled at the transcription level, with specific RNA transcripts present in regions of the flower primordia, which are affected by the specific genes (O‘maoiléidigh et al., 2014). As described in the ABC model for floral development, APETALA2 (AP2), AP1, AP3, PISTILIATA (PI) and AGAMOUS (AG) are essential components for regulating the development of various floral organs (Bowman et al., 1991; Thomson and Wellmer, 2019). Floral meristem identity genes regulate the spatially restricted patterns of the classical ABC model which includes AP3, PI, and AG (Weigel and Meyerowitz, 1994).

The time of flowering is controlled by several kinds of pathways, including photoperiod, gibberellin, autonomous, and vernalization (Boss et al., 2004; Freytes et al., 2021). These various floral-promoting signals regulate the expression of a common set of genes collectively referred to as flowering pathway integrators. Flowering pathway integrators activate floral-meristem identity genes and trigger the transition from the vegetative phase to the reproductive phase (Henderson and Dean, 2004; Komeda, 2004; Thomson and Wellmer, 2019). The chronological progression of MADS-domain transcription factor AG activation followed by the repression of WUSCHEL (WUS) is essential for the formation of reproductive floral organs and flower determinacy (Yan et al., 2016; Jha et al., 2020). It has been demonstrated that AG promotes the recruitment of TFL2/LHP1 to the stem cell maintenance gene in flower meristems (Liu et al., 2011), and directly or indirectly represses the expression of *WUS* (Lenhard et al., 2001).

Flowering is regulated by multiple flowering pathways that are controlled by environmental and endogenous signals. Recent evidence indicates that GA biosynthetic genes are induced by cold stress in dormant buds (Rinne et al., 2011) and that GA is closely involved with the regulation of anthesis in rosaceae species (Barros et al., 2012). In addition to GA, there are many hormones involved in the regulation of flowering. Auxin is very important to terminate the growth of meristem and promote flower meristem initiation (Cucinotta et al., 2021). For ethylene, Chen et al. found ETHYLENE RESPONSE FACTOR1 (ERF1), a key transcription factor of ethylene signaling, can delayed floral transition through direct inhibition of *FLOWERING LOCUS T* (*FT*) (Chen et al., 2021). Brassinosteroids (BR) as an important plant hormone has been linked to flowering in previous studies. Some BR biosynthesis and signaling transduction defect mutants showed delayed flowering (Li et al., 2010). Domagalska et al. found that BR could promote flowering by repression the expression of *FLOWERING LOCUS C* (*FLC*) (Domagalska et al., 2007). Recently, it was showed that BR downstream effectors BRASSINOSTEROID INSENSITIVE 2 (BIN2) and BRI1 EMS SUPPRESSOR1 (BES1) can interacts with FRIGIDA and ABSCISIC ACID-INSENSITIVE 3 (ABI3) to promote flowering, respectively (Hong et al., 2019; Khan et al., 2022). Photoperiod is another important factor to control plant flowering. Circadian system mediate photoperiodic regulation of day-length specific expression of *FT* is crucial for flowering time regulation (Song et al., 2013). Low-temperature is another important environmental factor, its signaling can be mediated by the C-repeat binding factor (CBF)/DREB1 family of transcription factors (Barros et al., 2012; Li et al., 2020). Low temperature signaling pathways were also studied on the cold-regulated (COR) genes, which play a fundamental role in stress tolerance, and determined to be targets for CBF TFs (Hwarari et al., 2022). The *Arabidopsis* CSPs which contain an N-terminal cold shock domain have been shown to have an important functional role in flowering development (Nakaminami et al., 2009; Juntawong et al., 2013). CSPs are associated with abiotic stress responses where AtCSP1-AtCSP3 genes are highly induced by cold stress (Karlson and Imai, 2003). Although regulation of cold acclimation and dormancy in perennial plants has received increasing attention in recent years, the role of the molecular pathways connected with low-temperature signaling is still poorly understood.

The transition of vegetative phase to reproductive phase is a critical decisive element for flowering plants. In tobacco, delayed flowering improves both biomass and biomass digestibility. The vast experience in cultivating tobacco garnered over the years suggests that flowering time is an important biomass trait and could greatly affect the quality of tobacco leaves. Our study demonstrated that cold conditioning in the seedling stage may lead to an early flowering transition of tobacco. While recent studies have also illustrated that cold induces notable effects on tobacco photosynthesis and secondary metabolism (Huang et al., 2016) little has been accomplished in exploring the molecular mechanism for early flowering transition based on induced cold condition. Plant breeding in order to influence cold requirements and develop improved freeze tolerant cultivars is considered to be long-term solutions to mitigate winter chill, decrease freezing damages, and secure deciduous fruit production (Zhang et al., 2019). In this study, two tobacco varieties Xingyan 1 (XY1) and Yunyan 85 (YY85) were thermally treated with 12°C as cold stress. Comparative transcriptomic and functional analysis was performed to evaluate the expression of genes involved in flower pathway and reveal the inherent molecular mechanism for regulating the flowering transition.

## Materials and methods

### Plant material and cold-stress treatment

Tobacco seeds of YY85 and XY1, incubated in water until germination, were sown into seedbeds. The seedbeds were then transplanted into potted containers and placed in a light incubator with a constant temperature of 28°C and relative humidity of 70-80%. The photoperiod was defined as 12 h with white light (1500 lx) and 12 h without light in one day. When sixth true leaf appeared, some of the seedlings of YY85 and XY1 were then transferred into another light incubator with temperature set to 12°C as the cold stress treatment. The remaining seedlings were considered as a control group. After 10 days, stem tips of the seedlings from cold and control groups were collected for analysis and three independent biological replicates were used in this study. The 10 days cold treated plants were then transplanted into normal growth environments.

### Microscopy analysis

The stem tip tissues used for light microscopy were harvested from the 25- and 30-day-old plants after transplantation and temperature treatment (12°C and 28°C, as previously described). Stem tips (about 5 cm long) were cut down and placed into a medical syringe with a length of at least 15 cm to exhaust air in the stem tip. The stem tips were then fixed in FAA fixation solution for 48 h. The anatomical structure of the meristem tips was then observed *via* an SZX12 stereomicroscope (Olympus Corp., http://www.olympus.co.jp/en/) at ×50 magnification and pictures were captured.

### RNA preparation

Total RNA was extracted from 100 mg of tobacco leaves using the Plant RNA Isolation Kit (Watson Biotechnologies, Inc., Shanghai, China). DNase digestion was performed using RNase-free DNase I (TaKaRa) in order to remove any residual DNA. The concentration and quality of RNA were determined using a NanoDrop ND-2000 Spectrophotometer (Thermo Scientific, Waltham, MA, USA) and an Agilent 2100 BioAnalyzer (Agilent Technologies, Cheadle, UK). The RNA samples from the same cultivated region were then equivalently pooled together for microarray hybridization and qRT-PCR analysis.

### Transcriptomic analysis

Gene expression profiles of tobacco leaves were assessed *via* Agilent Tobacco Gene Expression Microarrays (G2519F). cRNA labeling, hybridization and microarray processing were carried out at CapitalBio Corporation (Beijing, China) and labeling reactions were carried out using a CapitalBio cRNA Amplification and Labeling Kit (CapitalBio Corp.) in the presence of a fluorescent dye (Cy3-dCTP). All samples were labeled with Cy3 in this study. In order to rule out bias attributed to the dye, the sample labeling with these dyes was reversed (Yu et al., 2010). Hybridization was performed at 42°C for 16 h in a CapitalBio BioMixer II Hybridization Station (CapitalBio Corp.). Microarray slides were scanned with a LuxScan 10KA confocal laser scanner (CapitalBio Crop.) at 535 nm for Cy3 and 625 nm for Cy5 after washing. The obtained images were then evaluated with LuxScan 3.0 software (CapitalBio Corp.) which employs the LOWESS method (locally weighted scatter plot smoothing regression) (Yang et al., 2002) to minimize differences of dye incorporation efficiency in a two-channel microarray platform. Spots with fluorescence signal intensities of < 800 U (after subtracting the background in both channels) were regarded as empty spots and not analyzed further.

### Differentially expressed gene identification

Differential expression analysis was performed in comparing samples of the cold treatment group and control group. The raw fluorescence signal intensity of each spot was normalized to represent the expression level of one gene. The criteria used to identify differentially expressed genes (DEGs) were a fold change of either < 0.5 or > 2 and a p-value of < 0.05 when a comparison was made between samples.

### Functional annotation

Due to the limited annotation of the Agilent Tobacco Gene Expression Microarray, enhanced function annotations for probe sets were established from the best BlastX hit of the *Nicotiana tabacum* genome (https://solgenomics.net/organism/Nicotiana_tabacum/genome) with an e-value cutoff of 1e^-3^. KEGG enrichment analysis of DEGs was performed using KEGG database, and gene functions were categorized using MapMan bin codes (http://mapman.gabipd.org/) (Usadel et al., 2005). The predication for identified genes derived from tobacco was performed by transferring annotations to the tomato and *Arabidopsis* genome in consideration of orthologous genes. Pathway mapping and Gene Ontology (GO) enrichment analysis of identified genes was performed using the Kyoto Encyclopedia of Genes and Genomes (KEGG) database (http://www.genome.jp/kegg/) and the gene ontology resource (http://geneontology.org/) (Kanehisa and Goto, 2000).

### Cluster analysis of differentially expressed genes

Cluster analysis of DEGs was performed using Cluster 3.0 software (version 3.0; http://bonsai.hgc.jp/~mdehoon/software/cluster/) (De Hoon et al., 2004).

### Quantitative real-time PCR

Total RNA was isolated from stem tips using the method(s) described above. RNA was reverse-transcribed using a cDNA synthesis kit (Promega, Madison, WI, USA) by following the manufacturers protocol. The qRT-PCR was performed using an EvaGreen qPCR MasterMix (Applied Biological Materials, Richmond, BC, Canada) on a MyiQ2 two-color real-time PCR detection system (Bio-Rad, Hercules, CA, USA). The reaction conditions were as follows: 95°C for 600 seconds followed by 35 cycles of 95°C for 15 seconds and then 60°C for 60 seconds. Gene expression was normalized using *actin* as an internal control. Three biological replicates were performed for each sample. The primers used (**Table S1**) were designed using Primer Premier 5.0 software (Premier Biosoft International, Silicon Valley, CA, USA) and the relative quantification method (2-ΔΔCt) was utilized to evaluate quantitative variation between the different treatments (Livak and Schmittgen, 2001).

### Measurement of bioactive BR level

Enzyme-linked immunosorbent assays (ELISAs) for measuring 24-epicastasterone and related brassinolide (BL) analog (with detection ranges of 0.005 to 50 pmoles) was used in this study. A portion of the stem tip samples were ground in liquid nitrogen using a pre-cooled mortar and pestle and the resulting powder was then mixed with 2 mL of 80% methanol. The mixture was incubated for 4 h at 4°C and then centrifuged for 15 min at 1200 × g. After the supernatant and precipitate were separated, the precipitate was extracted again with 1 mL methanol and the combined supernatant was then dried *via* nitrogen using a Nitrogen Blowing Apparatus (YGC-36, Baojing, Zhengzhou, China). The residue adhered inside the tube was BR extraction. The steps for BR level measurement were performed following the manual’s instruction for the ELISA Kit (Beinongtianyi, Beijing, China), with the BR content in the fresh stem tips expressed as ng/g FW.

### Generation of *NtBRI1* overexpression lines and *NtBRI1* mutant plants

According to the gene sequence of *NtBRI1* (*Nitab4.5_0005374g0010*), *NtBRI1* amplification primers (NtBRI1-F: GGATCATCATGAACCTCACAAGTGCTA, NtBRI1-R: ACTAGTTCATAGGTGTTGCTCAGCTCAT) were designed, and BamH I and SPE I digestion sites were introduced into the primers. Then, *NtBRI1* was amplified by PCR and introduced into pCAMBIA2301 binary vector. To generate the *NtBRI1* CRISPR/Cas9 vector, one target sequence was selected according to a web tool (http://chopchop.cbu.uib.no/), and sgRNA was generated by using overlapping PCR. And then the sgRNA was subcloned into the BamHI site of pCACas9 binary vector. The confirmed plasmids were introduced into *Agrobacterium tumefaciens* strain GV3101 for subsequent tobacco genetic transformation. Finally, a main cultivated tobacco variety (Honghua Dajinyuan, HD) was used to generate *NtBRI1* overexpression plants and *NtBRI1* mutant plants.

### Statistical analysis

SPSS statistical software (version 22.0; IBM, Armonk, NY, USA) was used in the statistical analysis in this study. Statistical significance was evaluated by either the Student’s *t*-test when only two groups were compared or by one-way ANOVA followed by Tukey’s test when multiple groups comparisons. A p-value less than 0.05 was considered as statistically significant.

Data sets containing three independent biological replicates per sample were statistically analyzed. Principal component analysis (PCA) was performed using SIMCA-P software (Umetrics, Umea, Sweden) to assess the expression changes between samples and identify the transcriptomic changes involved in group discrimination.

## Results

### The effect of cold treatment on flower bud differentiation of XY1 and YY85 strains

**Figure 1:**
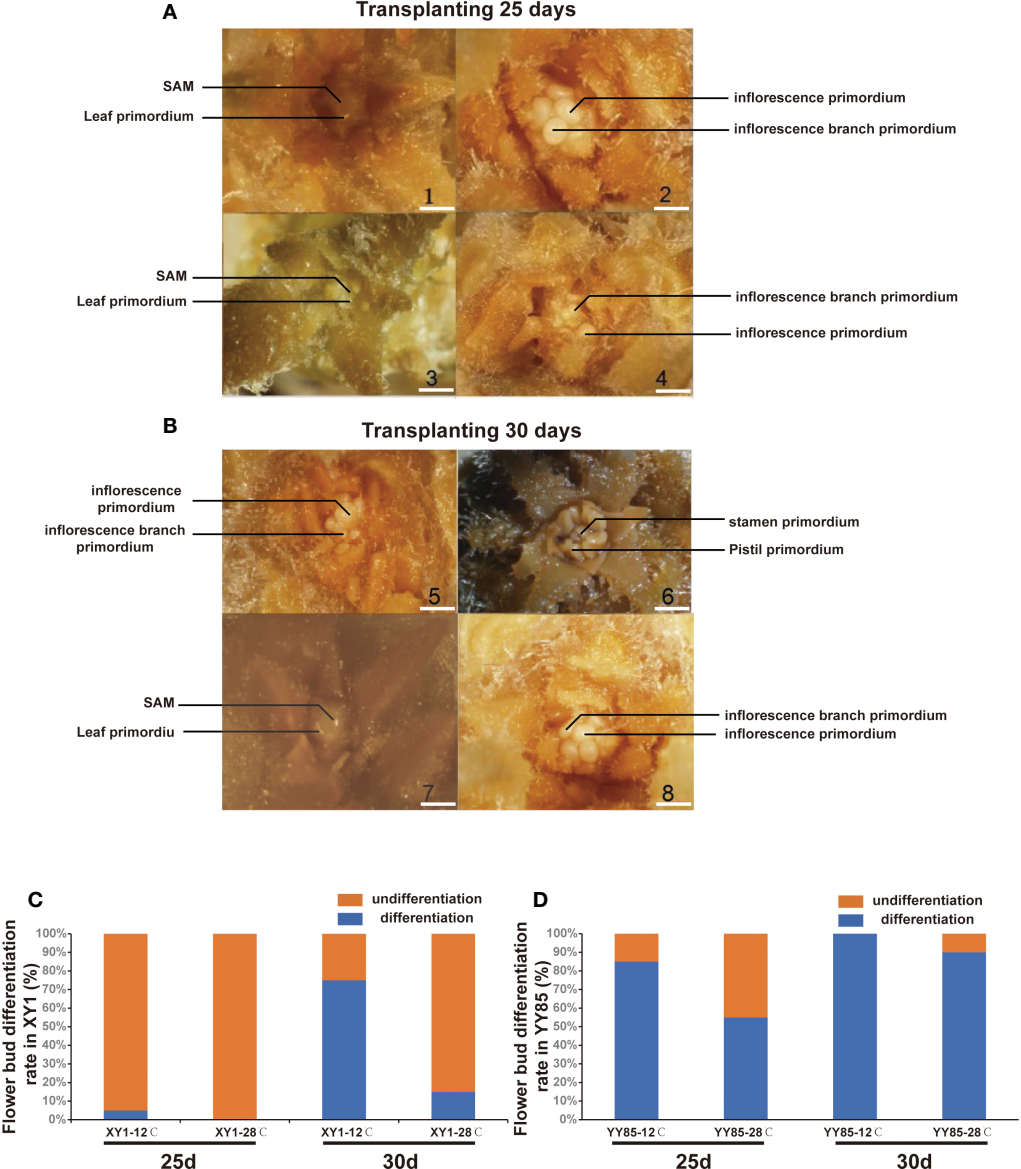
Microscope observation of cold stress induced flower bud differentiation. **(A)** and **(B)** Phenotype characteristic of tobacco stem tips under microscope. XY1 under 12°C treatment (1 and 5); YY85 under 12°C treatment (2 and 6); XY1 under 28°Ctreatment (3 and 7); YY85 under 28°C treatment (4 and 8). Bar = 1 mm. (SAM means shoot apical meristem). **(C)** and **(D)** The flower bud differentiation rate of XY1 and YY85 under cold-stress treatment. 20 samples of each treatment were collected for observation, and the experiment was repeated twice.

In investigating the effects of cold stress on flower bud differentiation, the seedlings of *N. tabacum* (XY1 and YY85) were treated with cold stress (12°C for 10 days). After a transplantation of 25 and 30 days, the stem tips were collected for microscopy analysis. Both XY1 and YY85 exhibited an earlier flower bud differentiation in the cold treatment group than that of control group (**Figure 1**). Compared to the control seedlings, XY1 and YY85 in the cold treatment group had advanced to the initiation stage of flower bud differentiation (ISFD) and calyx primordial formation stage (CPFS), respectively, when after being transplanted for 25 days (**Figure 1A**). After 5 days, the cold treatment group XY1 and YY85 had reached the CPFS and pistil primordial formation stage (PPFS), respectively, while those in the control group remained at the ISFD and CPFS, respectively (**Figure 1B**), and YY85 is more sensitive to cold treatment. After being transplanted for 25 days, more than 80% seedlings had been completed flower bud differentiation (**Figure 1C**). In contrast, only a few plants of XY1 have completed flower bud differentiation at the same time (**Figure 1D**). Field observation also showed that cold treatment group reached the budding stage earlier than that of control (**Figure S1A**). Consistently, the number of leaves in the low temperature treatment group decreased significantly compared with control, owing to early flower bud differentiation (**Figure S1B**). Based on the comparison between XY1 and YY85, it was clearly demonstrated that YY85 exhibited earlier floral transition and development than that of XY1, and it is more sensitive to cold stress in cold-induced early flowering.

### The comprehensive transcriptomic analysis of XY1 and YY85 under cold stress

To investigate the molecular mechanism of tobacco flower in response to the cold treatment, transcriptomic analysis was performed using Agilent Tobacco Gene Expression Microarrays. RNA was isolated from the stem tips of XY1 and YY85 that were grown at 28°C and 12°C, and cDNA was synthesized, labeled, hybridized, and analyzed. Principle component analysis (PCA) on the gene expression was performed to demonstrate the particularity of cold treatment samples. In **Figure S2A**, cold treatment samples of XY1 showed good separatatio from the control samples in PCA1 (60.6%) with a similar result found in samples of YY85. In the cold treatment group, samples of the two strains were not separated in either of the PCA quadrants; however, in the control group, XY1 and YY85 were significantly separated in PCA2 (18.0%). Furthermore, the PCA analysis also exhibited demonstrable repeatability in the replicated samples in XY1 and YY85. The DEGs were selected for further cluster analysis using cluster 3.0 software. A total of 7326 genes that were differentially expressed in response to the cold stress in the two tobacco strains were examined based on their expression values and then clustered into 2 groups (**Figure S2B**). Genes in cluster I were upregulated while genes in cluster II were downregulated in response the cold stress of the two tobacco strains. Cluster analysis performed between samples produced two sub-clusters, indicating a dramatic influence of cold stress on gene expression in stem tips of tobacco.

**Figure 2:**
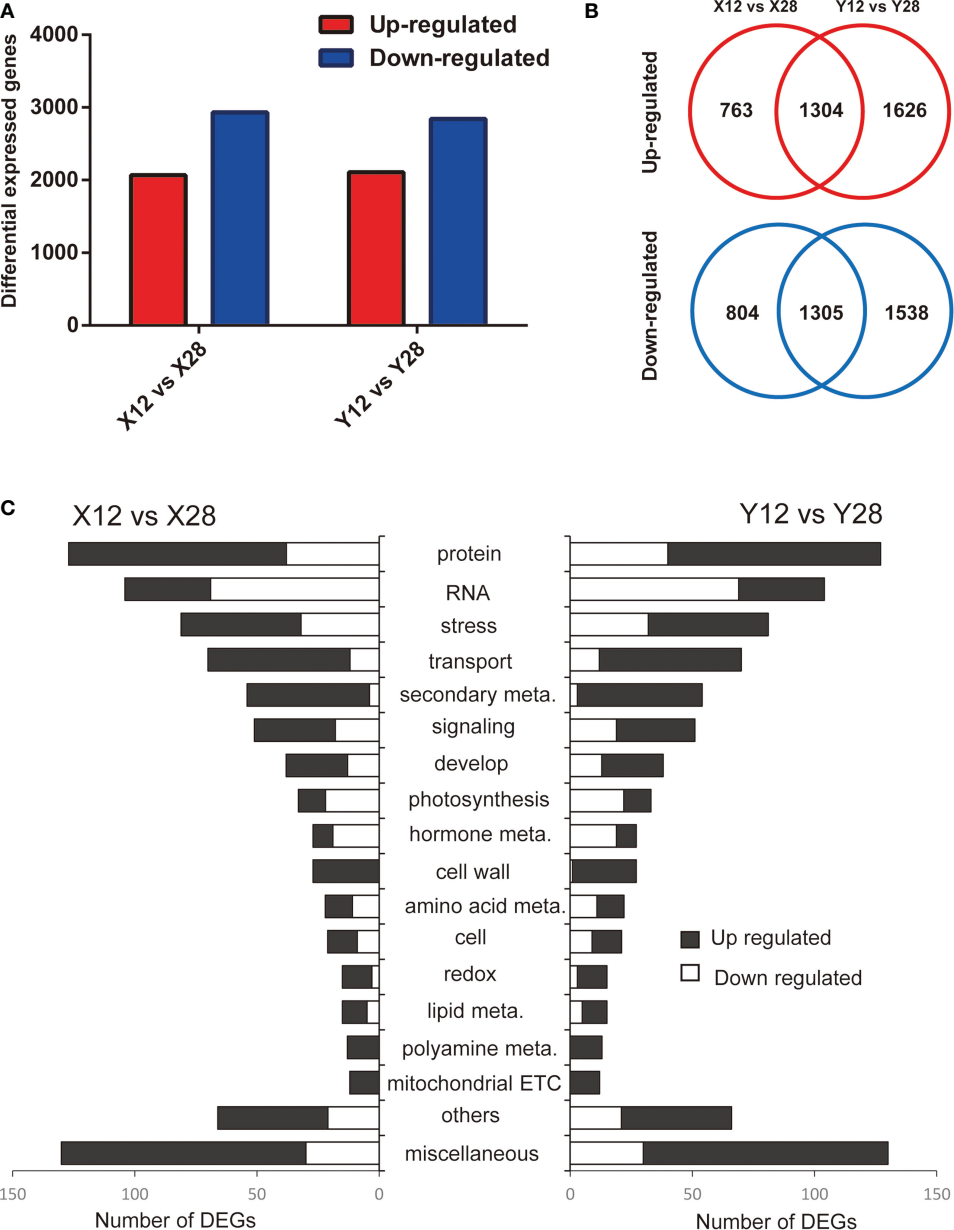
Overview of identified DEGs responding to cold treatment in different species of tobacco. **(A)** DEGs in XY1 and YY85 under cold stress treatment. **(B)** Up-regulated and down-regulated overlapped genes in XY1 and YY85 under cold stress treatment. **(C)** The DEGs were classified based on Mapman annotation.

In order to determine the mechanism by which cold affects stem tips of tobacco, expression levels of genes under cold treatment were compared with those of the control condition. A total of 4176 and 5773 genes were differentially expressed in response to the cold treatment in XY1 and YY85 (**Figure 2A**; **Table S2**), respectively, with 2609 DEGs commonly detected in both XY1 and YY85. Among the commonly detected DEGs, 1304 and 1305 DEGs were upregulated and downregulated in response to the cold stress, respectively (**Figure 2B**).

The multiple functions of DEGs were predicted based on comparisons with functional annotations of the *N.tabacum* genome and were classified using MapMan bin codes. The results of the functional analyses demonstrated that the DEGs exhibited an extremely similar functional distribution in both XY1 and YY85 when responding to cold treatment. The DEGs were mainly enriched in protein metabolism, RNA, stress, transport, and secondary metabolism. Genes involved in secondary metabolism, cell wall, redox, polyamine metabolism, and mitochondrial ETC were nearly all upregulated in response to cold treatment while the downregulated genes were mainly enriched in RNA, protein metabolism, stress, photosynthesis, and hormone metabolism (**Figure 2C**).

Genes related to the flowering pathway are listed in the Song and Chen study (Song and Chen, 2018). In order to analyze the expression of tobacco flowering genes in response to cold, the flowering pathway genes of DEGs in tobacco were list in **Table 1**. Genes associated with the MADS-box transcription factor family (AGL21, AP1, AGL67, AGL7, and AG), which is a key regulator of the transition into flowering and flower development, were all upregulated in both XY1 and YY85 in response to the cold stress. The upregulation of these genes indicates an advanced flower transition and development in tobacco under the cold treatment, with *HUA2* and *Flowering locus C* (*FLC*) genes acting as repressors in flower transitioning in *Arabidopsis* (Jali et al., 2014; Kinmonth-Schultz et al., 2021). It was demonstrated in this study that *HUA2* was downregulated in both XY1 and YY85 in response to the cold stress as well as *NtFLC* expression inhibition (determined by qRT-PCR) in the two tobacco strains when cold stressed (**Figure S3**).

**Table 1:** The differential expression of flowering pathway genes in tobacco under cold stress.

ProbeName	XY1	YY85	Accession	Description
A_95_P060245	down	down	BP134948	HUA2
A_95_P164432	up	up	FG151495	AGL21
A_95_P196857	up	up	TA16434_4097	AP1
A_95_P206422	up	up	TA18512_4097	AGL67
A_95_P245952	up	up	EB680458	AP1, AGL7
A_95_P292643	up	up	EB427886	AG

Phytohormones are crucial for plant flowering, and we sorted the hormone signaling related DEGs in our microarray data. A total of 113 genes were identified to be involved in five phytohormone signal pathway, including auxin, ethylene, abscisic acid (ABA), gibberellin acid (GA) and cytokinin (**Table S3**). Among them, auxin and ethylene had the most significant changes, with 42 and 41 differently expressed genes, respectively, including auxin transporters, receptors, auxin response factors and repressors, auxin response genes, and lots of ethylene-responsive transcription factors. Abscisic acid signal had 11 DEGs, cytokinin signal had 11 DEGs, and gibberellin signal was the fewest 8 DEGs. Of all 112 genes, 38 genes were up-regulated by cold stress, 72 genes were down-regulated by cold stress, and the expression of 2 genes showed different trends in XY1 and YY85. Most down-regulated genes were involved in auxin and ethylene signaling pathway. About 70% DEGs involved in auxin and ethylene signaling were down-regulated genes by cold stress.

Flower bud differentiation in stem tips is tightly linked to cell differentiation and proliferation. We found that a large number of genes related to cell division and cell cycle were differentially expressed under cold treatment, including many *cell division protein kinases* (*CDKs*), *cell division cycle* (*CDCs*) genes, *Cyclin B* and *Cyclin D* genes (**Table S4**). Furthermore, expansin proteins is crucial regulators of cell growth by modulating cell wall organization, and we found that many *expansin* genes were up-regulated by cold stress (**Table S4**). These data indicating that cold stress may reprogram cell differentiation, proliferation and growth of tobacco shoot apical meristem (SAM) to promote flower bud differentiation.

**Figure 3:**
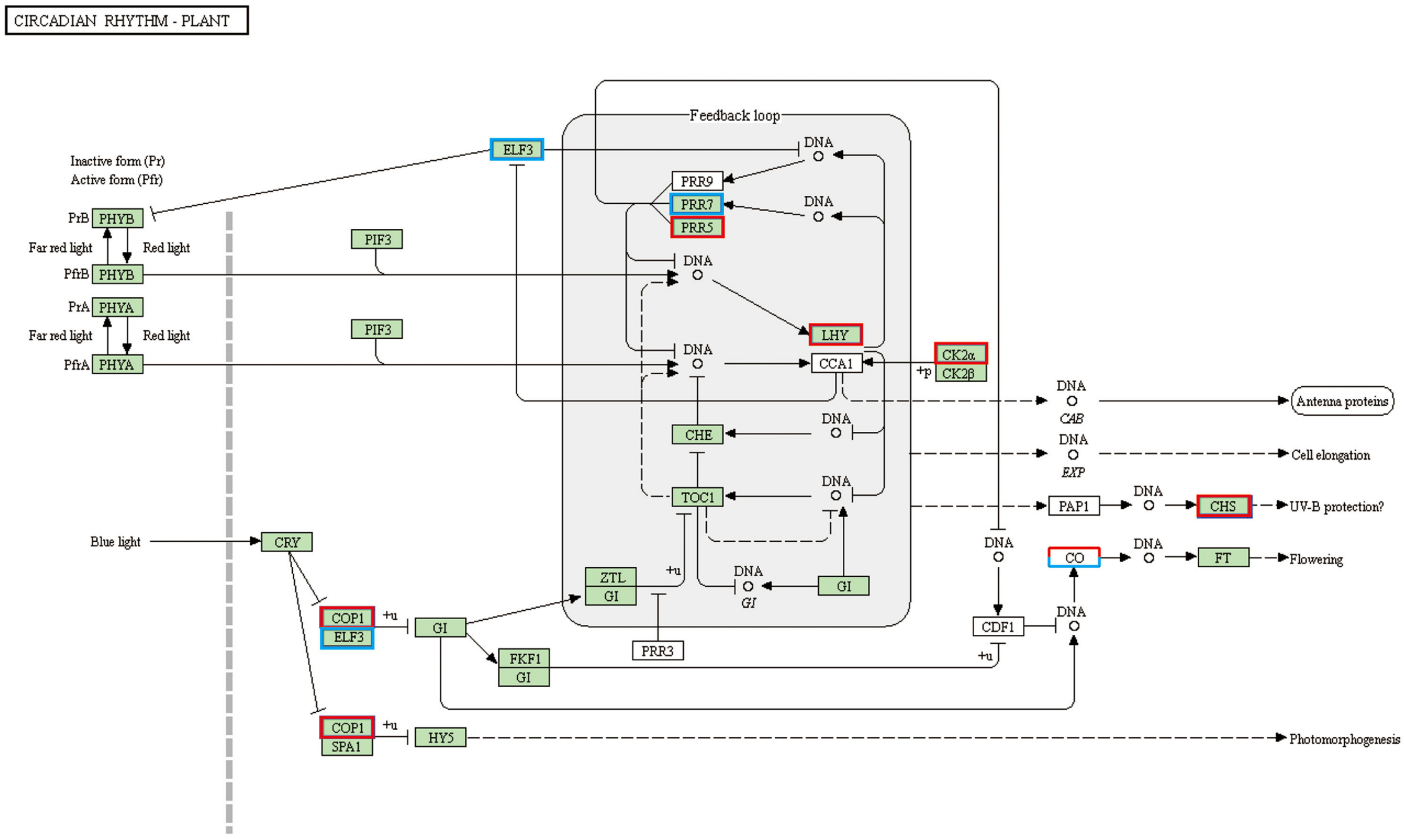
Integrated pathway mapping of circadian rhythm related DEGs. DEGs were selected based on the KEGG enrichment (Red indicates up-regulated and blue indicates down-regulated).

**Figure 4:**
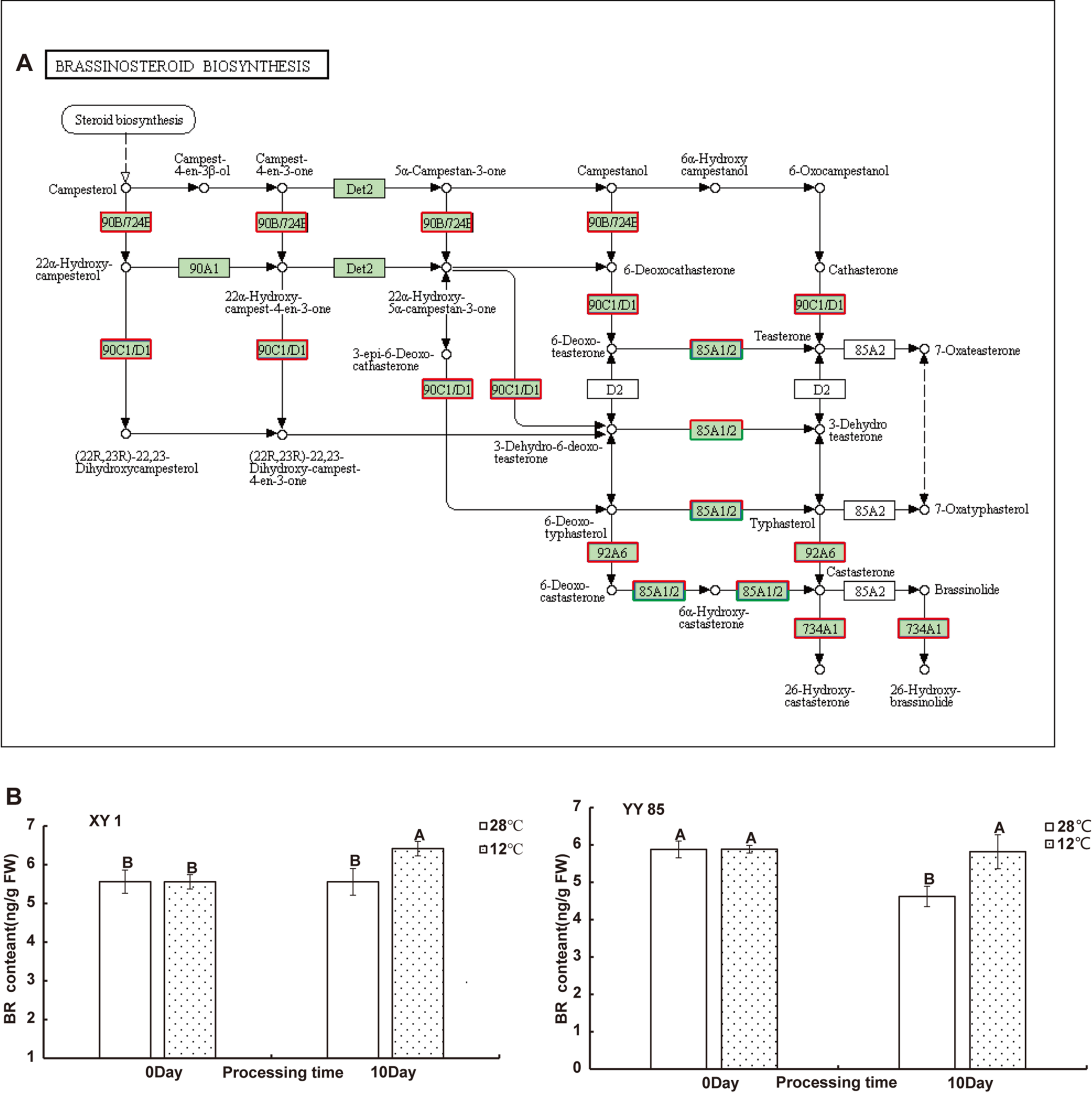
Integrated pathway mapping of BRs biosynthesis related DEGs. **(A)** DEGs were selected based on the KEGG enrichment (Red indicates up-regulated and green indicates down-regulated). **(B)** BR content in different species responding to cold treatment. Data are shown as the mean ± SD from three independent experiment replicates. Different letters indicated significant different (P<0.05, Tukey’s test). NS, no significant difference.

To further understand the function of DEGs affected by cold stress in tobacco, all detected genes were subjected to the KEGG and GO enrichment analysis (**Table S5**; **Figure S4**). Twelve and twenty enriched KEGG pathways were identifified as signifificant with Qvalues < 0.05 in XY1 and YY85, respectively (**Table S5**). An internal timing mechanism called the circadian clock is important for determining flowering by integrating multiple environmental signals (Freytes et al., 2021). KEGG enrichment data showed that 22 DEGs are enriched in the circadian system with a large number of up-regulated genes, including *COP1*, *LHY* and *CO* et al, indicating cold-induced early flowering is also closely related to circadian clock (**Figure 3** and **Table S7**). During the signifificantly altered KEGG pathways, some secondary metabolism pathway, starch and sucrose metabolism, amino acid metabolism and brassinosteroid biosynthesis are involved in tobacco response to cold stress. For brassinosteroid biosynthesis, ten brassinosteroid biosynthesis related genes were identifified with a signifificant change in their expression levels (with 9 up-regulated and 1 downregulated) in response to the cold stress (**Table S4**). The function and the expression of these DEGs are displayed in **Figure 4A**. In addition to brassinosteroid, we also found that many hormones synthesis related genes were differently expressed, including: auxin, zeatin-riboside, ethylene, abscisic acid, gibberellin and cytokinin (**Table S6**). These results indicated that cold stress promoted flower bud differentiation process by modulating multiple signal pathway in tobacco.

### BR signaling is crucial for cold induced early flowering in tobacco

The analysis of DEGs enriched in brassinosteroid biosynthesis pathway indicated that the brassinosteroid (BR) biosynthetic pathway may be affected by cold treatment, and in order to further analyze the relationship between cold stress and brassinosteroid in cold-induced tobacco flower bud differentiation, we measured the content of brassinosteroid in tobacco after cold stress treatment. BR was extracted from stem tips collected from tobacco under 28°C and 12°C conditions and the content was measured by using an ELISA technique to determine the level of BR in tobacco under cold stress treatment. The results indicate that BR contents in XY1 and XY85 tobaccos under cold treatment were significantly increased than those in control plants under normal condition (**Figure 4B**), indicating the mutual influence of cold treatment on BR content in tobacco plants. Furthermore, we found that the expression of *NtFLC* in tobacco was rapidly inhibited by BR, while *NtFLY* was not affected by BR in a short time treatment (**Figure S5**), suggesting that cold stress may suppress *FLC* expression through brassinosteroid signaling, leading to cold-inducing early flowering in tobacco.

**Figure 5:**
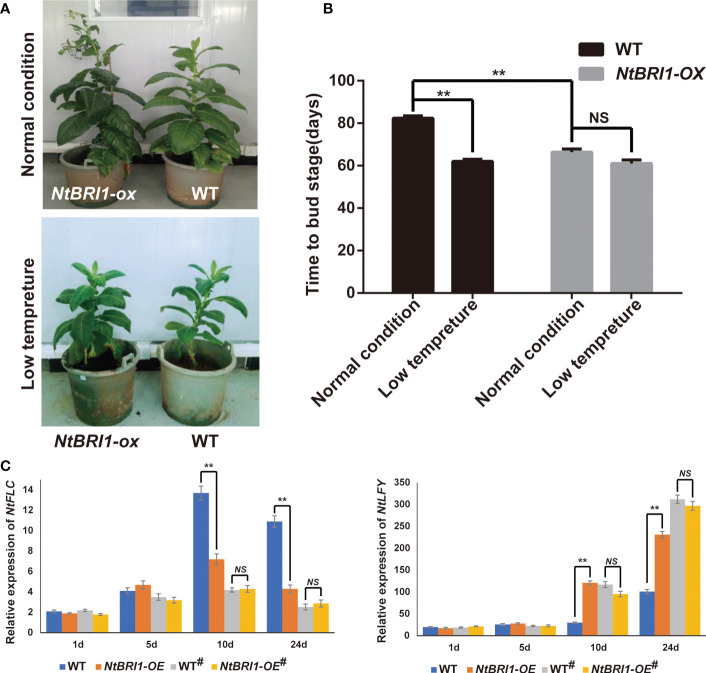
Overexpression of *NtBRI1* promote early flowering in tobacco. **(A)** The phenotype of *NtBRI1* overexpression plants. **(B)** The role of *NtBRI1* in regulating flowering time in tobacco. 5 samples of each treatment were collected for observation, and the experiment was repeated three times. **(C)** The expression of *NtFLC* and *NtLFY* in *NtBRI1* overexpression plants under normal or cold-induced conditions (# means cold treatment). The seedlings of WT and *NtBRI1* overexpression plants were treated with cold stress (12°C for 10 days, 16h light/8h dark), and then all plants were moved into the greenhouse (28°C, 16h light/8h dark). The samples were collected in 1d, 5d, 10d and 24d after cold treatment for qRT-PCR. Significant changes measured by Student’s t test (** means p < 0.01).

**Figure 6:**
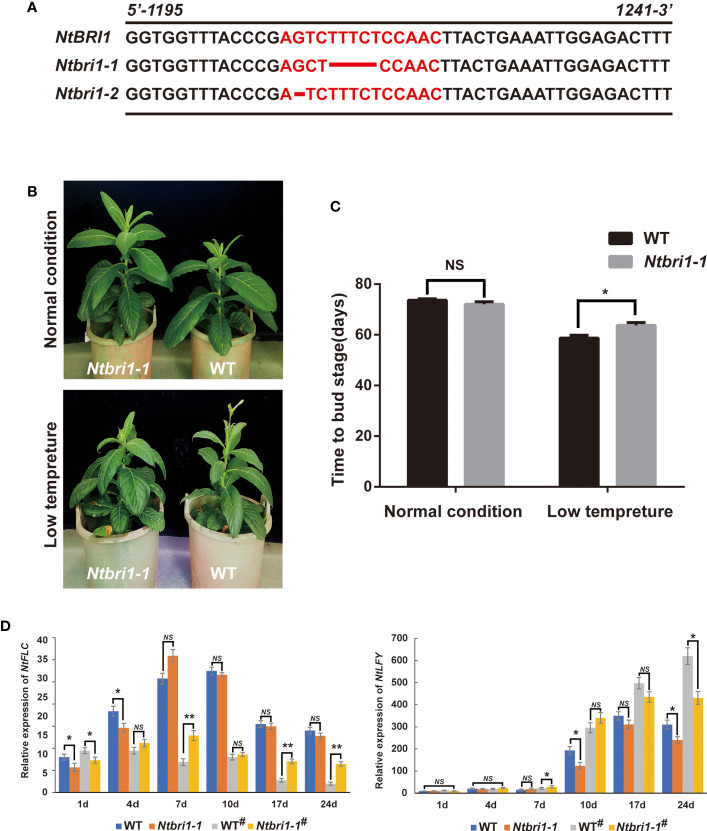
Cold induced early flowering is suppressed in *NtBRI1 mutant*. **(A)** The Crisper/Cas9 meditated gene edited site of *NtBRI1 in Ntbri1-1 and Ntbri1-2*. **(B)** and **(C)** The phenotype of *Ntbri1-1* plants in cold induced early flowering in tobacco. 5 samples of each treatment were collected for observation, and the experiment was repeated three times. **(D)** the expression of *NtFLC* and *NtLFY* in *Ntbri1-1* plants under normal or cold-induced conditions (# means cold treatment). The seedlings of WT and *Ntbri1-1* plants were treated with cold stress (12°C for 10 days, 16h light/8h dark), and then all plants were moved into the greenhouse (28°C, 16h light/8h dark). The samples were collected in 1d, 4d, 7d, 10d, 17d and 24d after cold treatment for qRT-PCR. Significant changes measured by Student’s t test (** means p < 0.01).

As a brassinosteroid receptor, BRI1 is essential for BR perception and signaling transduction by interacting with BAK1 to regulate plant growth and development in plants as well as in floral-induction (Li et al., 2002; Li et al., 2010; Nolan et al., 2019). And we found that the expression of *NtBRI1* was induced by BR and cold stress in tobacco (**Figure S6**). In order to understand whether cold-induced early flowering is dependent on BR signaling, we constructed *NtBRI1* overexpression lines (*NtBRI1-ox*). Under normal conditions, *NtBRI1-ox* exhibited earlier flowering than WT. While, *NtBRI1-ox* and WT showed no significant difference in flowering time under low temperature treatment (**Figures 5A, B**). qPCR data showed that the expression of *FLC* was significantly inhibited in the wild type plants under low temperature treatment. And the expression of *FLC* gene was at a low level in *NtBRI1-ox* whether treated with low temperature or not. On the other hand, low temperature could induce the expression of *LFY*, an essential transcriptional regulator that promotes the transition to flowering, in wild-type plants, but the expression of LFY in *NtBRI1-ox* is at a high level whether treated or not treated with low temperature (**Figure 5C**). Furthermore, the muta*nt of NtBRI1* was generated (*Ntbri1-1 and Ntbri1-2*), and several bases deletion in *NtBRI1* lead to early translation termination in *Ntbri1-1 and Ntbri1-2* (**Figure 6A**). Under normal conditions, *Ntbri1-1* and WT show similar flowering time. After cold treatment, both *Ntbri1-1* and WT exhibited early flowering, and *Ntbri1-1* flowering later than that of WT (**Figures 6B, C**). In addition, we found that the expression of *FLC* was significantly induced in *Ntbri1-1* compared with WT after cold treatment. And cold treatment induced the expression of *LFY* in *Ntbri1-1* was significantly lower than that of WT (**Figure 6D**). These results show that low temperature can regulate the expression of flowering related genes through BR signal, and *NtBRI1* participates in the regulation of cold induced early flowering in tobacco. Since the output of brassinosteroid signal mainly depends on the transcription factor of BES1 and BZR1, we analyzed the promoter of some flowering related DEGs (including circadian rhythm, hormone biosynthesis, hormone signal transduction, cell division, cell cyclin and expansin related DEGs) by using PlantPAN 3.0 web tools (http://plantpan.itps.ncku.edu.tw/) (Chow et al., 2019). Results showed that BES1 (CANNTG) and BZR1 (CGTGT/C) recognition motif exists on the promoter sequence of these genes (**Table S8**), indicating that brassinosteroid may affect the expression of these genes in the BES1 and BZR1 dependent manner.

## Discussion

### Early flowering was promoted in the cold treated tobacco plants

The transition from vegetative growth to flowering is of utmost importance, with environment cues influencing a plant’s flower transition through the strict genetic control (Quiroz et al., 2021). As described in the ABC model for floral development, AP2, AP1, AP3, PI, and AG are integral components for regulating development of various floral organs (Bowman et al., 1991). The floral meristem identity genes regulate spatially restricted patterns of the classical ABC model (Weigel and Meyerowitz, 1994; Irish, 2017). *AP1* is considered an expression marker for flowering that shows a quantitative relationship between expression level and flowering (Jaya et al., 2010). Cold treatment dismisses *AGL19* expression by activating AP1, thus resulting in flowering in *Arabidopsis* (Kim et al., 2013). Since AG is a master regulator that guides floral determinacy, *ag* mutations produced abnormal flower phenotypes (Bowman et al., 1989). In *Arabidopsis*, *AG* was activated during the early phase of flower development by the *LEAFY* and *WUSCHEL* genes (Busch et al., 1999; Lenhard et al., 2001; Lohmann et al., 2001). Early expression of *AG* was negatively regulated by AP2 and is distributed throughout third and fourth whorl organ primordia; however, it was proven that late *AG* expression is not directly dependent on AP2 activity (Bowman et al., 1991). *FLC* is the central regulator of flowering induction by vernalization, as there is a quantitative relationship between the duration of cold treatment and the extent of FLC activity downregulation. Furthermore, HUA2 acts as a repressor with *FLC*, as the missing expression of *HUA2* in the *hua2* mutant led to a significant decrease in *FLC* expression, but with no influence on *AG* expression (Cheng et al., 2003). In this study, a series of flowering pathway related genes (including *AP1* and *AG*) were shown to be upregulated while *FLC* and *HUA2* were downregulated in the stem tips of tobacco in response to the cold stress, indicating that cold stress induces events of floral transition and promote the flowering pathway.

### Cold induced early floral transition through regulation of the circadian system related genes and multiple hormones signaling pathways

Recent microarray analysis reveals that the cold response of plants was controlled by the circadian clock (Nakaminami et al., 2009). In this study, many circadian system related genes were identified and most of them were showed significantly increased expression in response to the cold stress (**Figure 6**; **Table S7**), indicating that cold may induce the floral transition by up-regulating the expression of circadian system related genes in tobacco. In further investigating the molecular mechanism of the regulation of early flora transition by cold, the DEGs were analyzed and showed that the ARR related genes exhibited positive expression in both XY1 and YY85. ARR (APRR) genes are important components of the circadian clock mechanism that regulates the diverse biological processes, including the control of flowering and abiotic stress response (Liu et al., 2015). PRR7, PRR9, and TOC1 have been shown to be transcriptional repressors involved in the transcriptional-translational feedback loops in the circadian network and can repress the clock components CCA1 and LHY when expressed at dawn (Nakamichi et al., 2010; Gendron et al., 2012). The CCA1 and LHY, in turn, can activate the expression of *PRR9* and *PRR7* as well as repress the expression of *TOC1* (Farré et al., 2005), and the Myb transcription factors, including CCA1 and LHY, act as activators for *TOC1* (Rawat et al., 2011). The constituted core feedback loop by CCA1, LHY, and TOC1 elicits a dramatic effect on the CO-FT pathway and regulates short vegetative phase (SVP) genes, with SVP represses the expression of the floral pathway genes by interacting with FLC in vegetative tissue (Li et al., 2008). In our study, the circadian clock related the genes *COP1*, *LHY*, and autonomous pathway related genes such as *CO* were significantly regulated by cold stress, suggesting a close relationship of cold and circadian system in tobacco flower bud differentiation (**Figure 6**; **Table S7**).

Floral transition is known to be regulated by multiple interacting endogenous hormone signaling and environmental cues. In this study, we found that a large number of hormone synthesis and signal transduction pathways related genes were differentially expressed under cold treatment, among which changes of auxin and ethylene signaling were the most obvious. Auxin can promote floral meristem initiation by modulating the expression of *LFY* (Cucinotta et al., 2021). Under cold treatment, we found that the expression of *NtLFY* was significant induced, suggesting that auxin may be involved in cold induced *NtLFY* expression to promote floral meristem initiation. In a previous study, Ogawara et al. showed that ethylene signaling is involved in regulation transition from vegetative growth to flowering in *Arabidopsis* (Ogawara et al., 2003). In our data, *EIN2* and *EIL3*, two key components of ethylene signal, are significantly up-regulated by cold stress, implying ethylene signaling was activated by cold stress. GA is generally understood to be one of the critical regulators in mediating the flowering (Jung et al., 2012). Recent evidence indicates that GA biosynthetic genes are induced by cold stress in dormant buds (Rinne et al., 2011); however, the detailed regulation mechanism in mediating the flowering in the cold treated plants were remain unclear.

In addition to playing a crucial role in flower bud differentiation, these changed hormone signals may also take part in tobacco response to cold stress. Usually, cold stress could induce the accumulation of ABA in plants, leading to the closure of plant stomata to help plants adapt to low temperature environment (Agurla et al., 2018). GAs induced degradation of DELLA repressor protein is also involved in cold responses. Recently, Lantzouni et al. showed that DELLAs could interact with GROWTH REGULATORY FACTOR (GRF) to regulate plant growth under cold stress (Lantzouni et al., 2020). Under cold stress, the biosynthesis and signal transduction of multiple hormones have changed, suggesting that the change of multiple hormone signals induced by cold stress may play two roles at the same time, including flower bud differentiation and cold stress tolerance.

### The brassinosteroid signaling regulated the cold induced early flowering

BRs are a class of steroidal hormones essential for plant growth and development (Kim and Russinova, 2020). And as biologically active agents, BL and other BRs have since been identified and widely recognized as a major group of plant growth regulators (Bajguz and Tretyn, 2003; Zullo and Kohout, 2004). In this study, the KEGG enrichment of DEGs demonstrated that the brassinosteroid biosynthesis pathway exhibited a very positive response to the cold stress, with phytochemical measurement confirming the increase of BL accumulation in the two cold treated tobacco strains (**Figure 3**). Although there is limited information regarding the function of BRs in regulation of flowering transition, the role of BRs in a flowering physiological study was investigated. Domagalska showed that the mutations impaired in BR biosynthesis exhibited typical phenotype of delayed flowering in *Arabidopsis* (Domagalska et al., 2007). As Li et al. reviewed, there may be multiple regulation relationships between BRs and the flowering time (Li et al., 2010).

One possible relationship is that BRs regulate the floral transition through the circadian system given the fact that it has been shown that BRs shortened the period of circadian rhythm of chlorophyll a/b binding protein (CAB), cold and circadian-regulated 2 (CCR2), and circadian clock associated 1 (CCA1) under both continuous white light and dark (Hanano et al., 2006). As we understand, the circadian clock is an endogenous mechanism that regulates metabolism and physiology in plant life. The circadian system displays dramatic effects on the flowering time by regulating the *CO* and *FT* genes (Suárez-López et al., 2001; Yanovsky and Kay, 2002). The secondary relationship is that BRs regulate the flowering time by mediating the expression of *FLC* by the BRI regulation pathway. It has been demonstrated in *Arabidopsis* that BR signaling promotes flowering by increasing the repression level of the *luminidependens* (*LD*), *FCA*, and *FLC* genes that are in the autonomous pathway (Domagalska et al., 2007). Another link between floral induction and BR signaling is the interaction between bri1-ems-supressor 1 (BES1) and early flowering 6/relative of early flowering 6 proteins. In Yu’s study, the BES1 was demonstrated to recruit ELF6 and REF6 to regulate the autonomous genes in *Arabidopsis* (Yu et al., 2008). Promoter analysis also showed that some BES1/BZR1 recognition motifs existed in DEGs, suggesting that these genes may be regulated by BES1/BZR1. In this study, we identified that BR signaling pathways may respond to the cold stress contributed to the apparent expression of related genes in these pathways, especially the *NtBRI1* genes. BL accumulation first responded to the cold stress in the stem tips of tobacco, and then the BR signaling regulated flowering related genes, thus leading to phenotypic characteristics of early floral transition.

## Data availability statement

The Microarray data are available in the Gene Expression Omnibus (GEO) database (www.ncbi.nlm.nih.gov/geo) under the accession number GSE227885.

## Author contributions

XD and YZ contributed to the study conception and design. LX, XZ and GJ performed the experiments. XX and MR analyzed the data. XD, JZ and KD wrote the paper, and all authors commented on previous versions of the manuscript. All authors read and approved the final manuscript. All authors contributed to the article and approved the submitted version.

## Supplementary material

Supplementary Figure 1 The phenotype of XY1 and YY85 plants in cold induced early flowering in tobacco. **(A)** The time to bud stage of XY1 and YY85 under cold treatment. **(B)** The number of leaves in XY1 and YY85 under cold treatment. 5 plants of each treatment were collected for data analysis, and the experiment was repeated twice.

Supplementary Figure 2 Microarray expression profile analysis of tobaccos responding to chill condition in different species. **(A)** PCA plot of microarrays (X12 means XY1 under 12°C treatment; X28 means XY1 under 28°C treatment; Y12 means YY85 under 12°C treatment;Y28 means YY85 under 28°C treatment). **(B)** Heat map and cluster of microarrays. The color bar represents gene expression level (FPKM). Three independent biological replicates were used in this analysis.

Supplementary Figure 3 qRT-PCR of *NtFLC* gene in different species responding to cold treatment. The seedlings of XY1 and YY85 plants were treated with cold stress (12°C for 10 days, 16h light/8h dark), and then all plants were moved into the greenhouse (28°C, 16h light/8h dark). The samples were collected in 0d, 4d, 7d, 10d, 17d and 24d after cold treatment for qRT-PCR (# means cold treatment). Data are shown as the mean ± SD from three independent experiment replicates. Significant changes measured by Student’s t test (* means p < 0.05 and ** means p < 0.01).

Supplementary Figure 4 Top 20 GO and KEGG enrichment of DEGs in XY1 and YY85 under cold treatment.

Supplementary Figure 5 qRT-PCR of *NtFLC* and *NtLFY* genes in responding to BR treatment. The seedlings of HD plants were grow in the greenhouse (28°C, 16h light/8h dark). When sixth true leaf appeared, plants were treated with brassinolide, and samples were collected in 0h, 2h, 4h, 6h and 8h after cold treatment for qRT-PCR. Data are shown as the mean ± SD from three independent experiment replicates. Significant changes measured by Student’s t test (* means p < 0.05 and ** means p < 0.01).

Supplementary Figure 6 qRT-PCR of *NtBRI1* in responding to BR treatment and cold stress. For BR treatment, the seedlings of HD plants were grow in the greenhouse (28°C, 16h light/8h dark). When sixth true leaf appeared, plants were treated with brassinolide, and samples were collected in 0h, 2h, 4h, 6h and 8h after cold treatment for qRT-PCR. For cold stress treatment, the seedlings of HD plants were treated with cold stress (12°C for 10 days, 16h light/8h dark), and then all plants were moved into the greenhouse (28°C, 16h light/8h dark). The samples were collected in 1d, 4d, 7d, 10d, 17d and 24d after cold treatment for qRT-PCR (# means cold treatment). Data are shown as the mean ± SD from three independent experiment replicates. Significant changes measured by Student’s t test (* means p < 0.05 and ** means p < 0.01)
