# Supplementary figures and images for: Transcriptome and functional analysis revealed the intervention of brassinosteroid in regulation of cold induced early flowering in tobacco
Source: Front Plant Sci. 2023 Mar 31;14:1136884. doi: 10.3389/fpls.2023.1136884 (PMC10102362; doi:10.3389/fpls.2023.1136884)

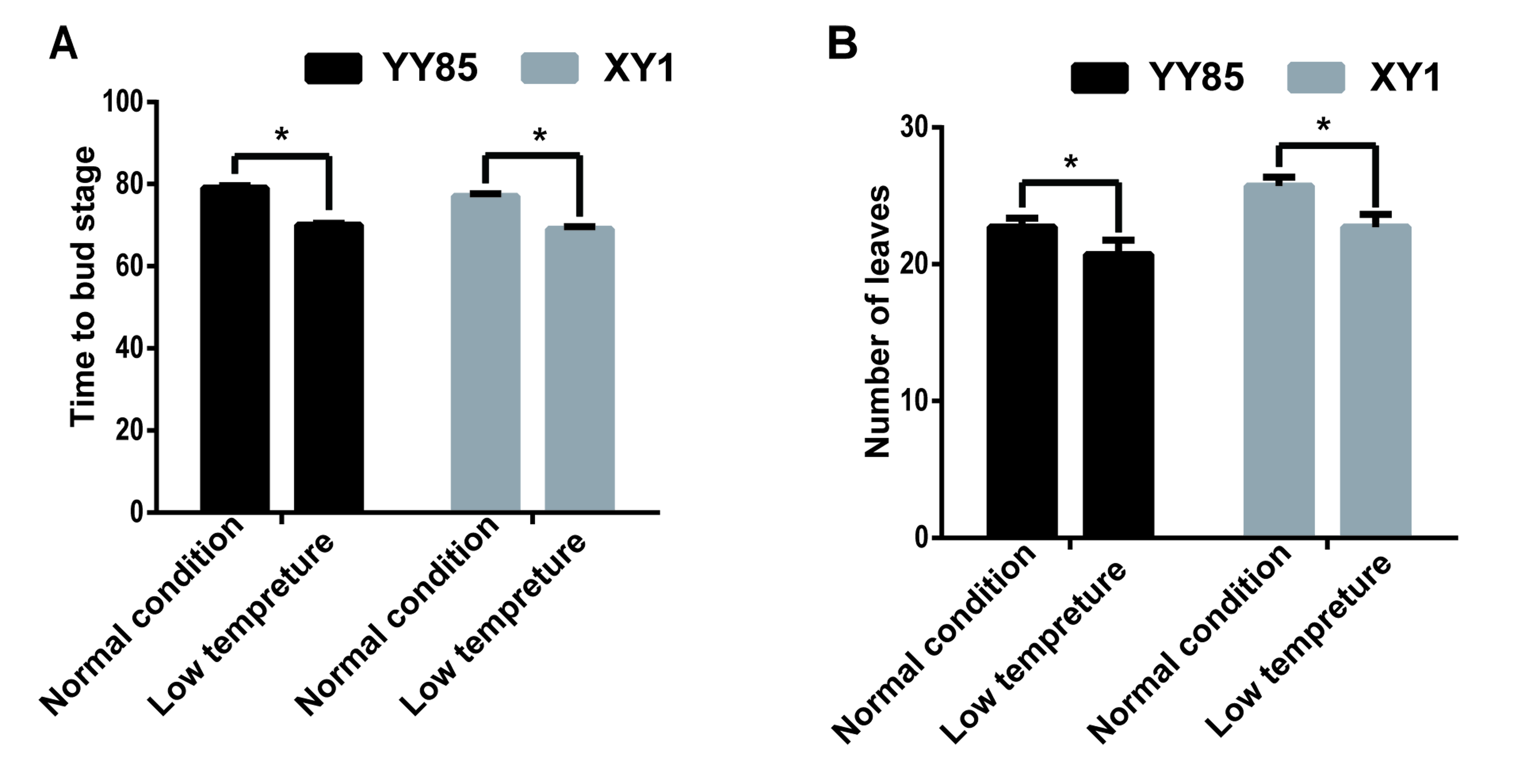

Supplement: Supplementary Figure 1 — The phenotype of XY1 and YY85 plants in cold induced early flowering in tobacco. (A) The time to bud stage of XY1 and YY85 under cold treatment. (B) The number of leaves in XY1 and YY85 under cold treatment. 5 plants of each treatment were collected for data analysis, and the experiment was repeated twice. [file Image_1.jpeg]

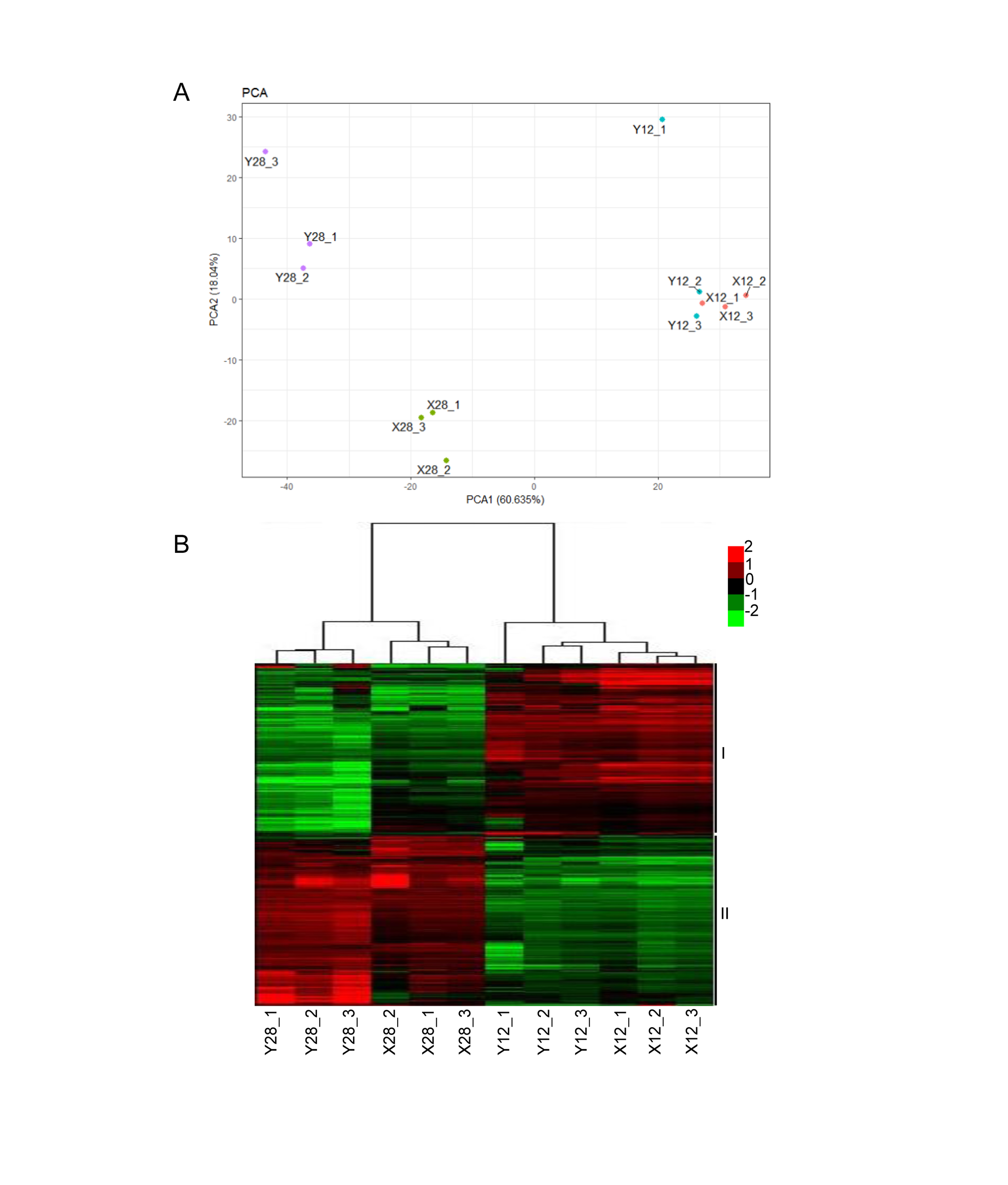

Supplement: Supplementary Figure 2 — Microarray expression profile analysis of tobaccos responding to chill condition in different species. (A) PCA plot of microarrays (X12 means XY1 under 12°C treatment; X28 means XY1 under 28°C treatment; Y12 means YY85 under 12°C treatment;Y28 means YY85 under 28°C treatment). (B) Heat map and cluster of microarrays. The color bar represents gene expression level (FPKM). Three independent biological replicates were used in this analysis. [file Image_2.jpeg]

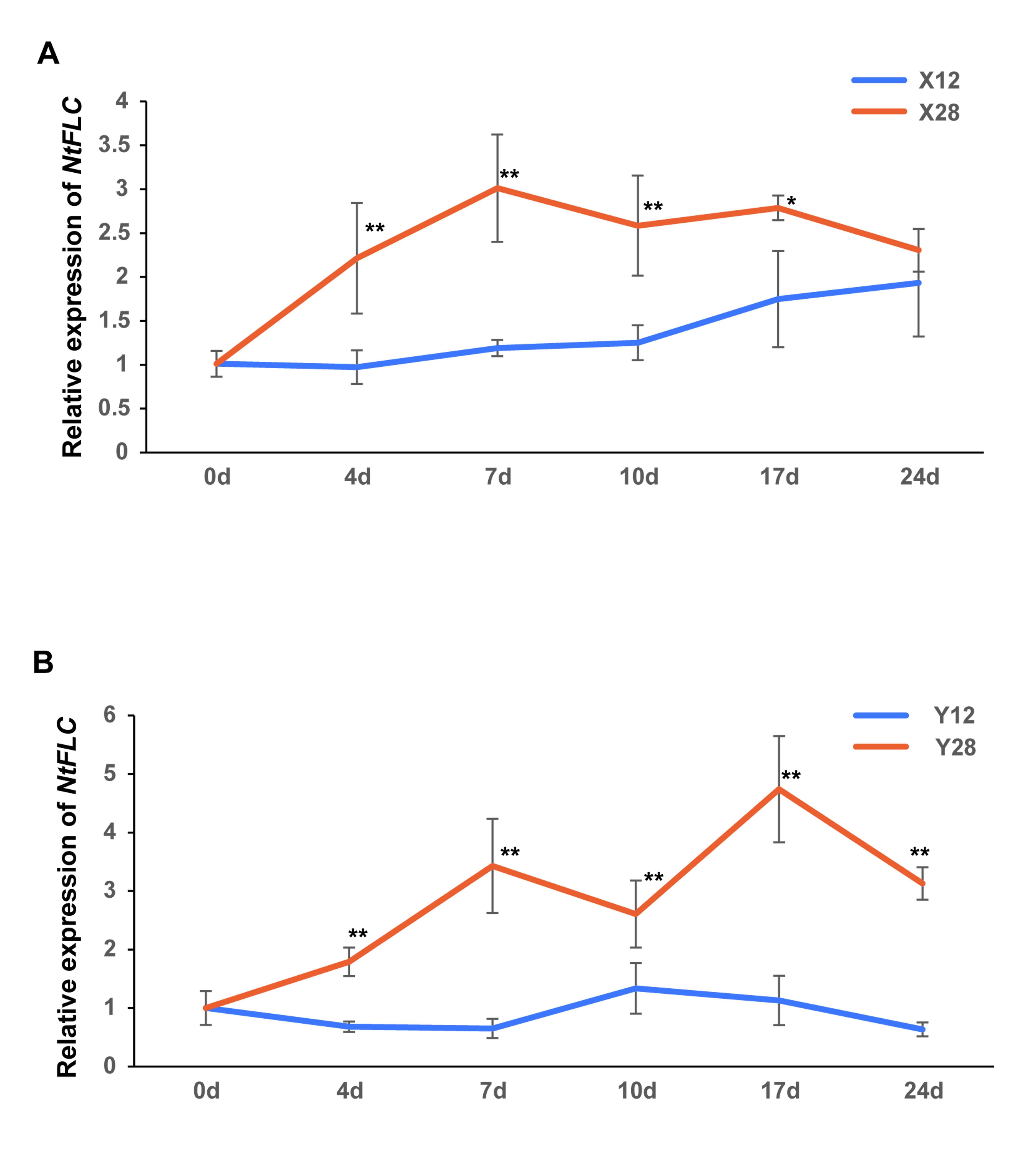

Supplement: Supplementary Figure 3 — qRT-PCR of NtFLC gene in different species responding to cold treatment. The seedlings of XY1 and YY85 plants were treated with cold stress (12°C for 10 days, 16h light/8h dark), and then all plants were moved into the greenhouse (28°C, 16h light/8h dark). The samples were collected in 0d, 4d, 7d, 10d, 17d and 24d after cold treatment for qRT-PCR (# means cold treatment). Data are shown as the mean ± SD from three independent experiment replicates. Significant changes measured by Student’s t test (* means p < 0.05 and ** means p < 0.01). [file Image_3.jpeg]

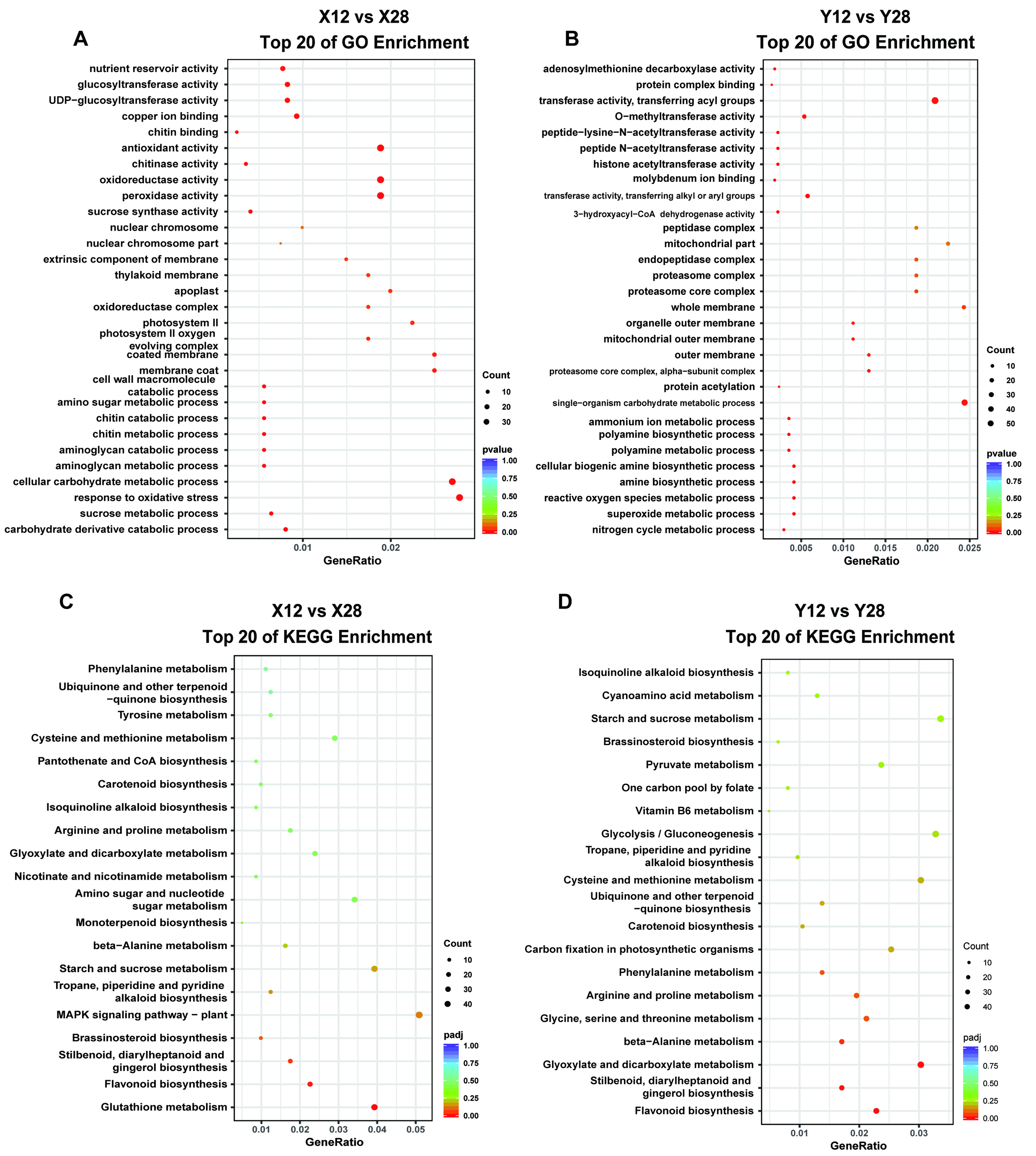

Supplement: Supplementary Figure 4 — Top 20 GO and KEGG enrichment of DEGs in XY1 and YY85 under cold treatment. [file Image_4.jpeg]

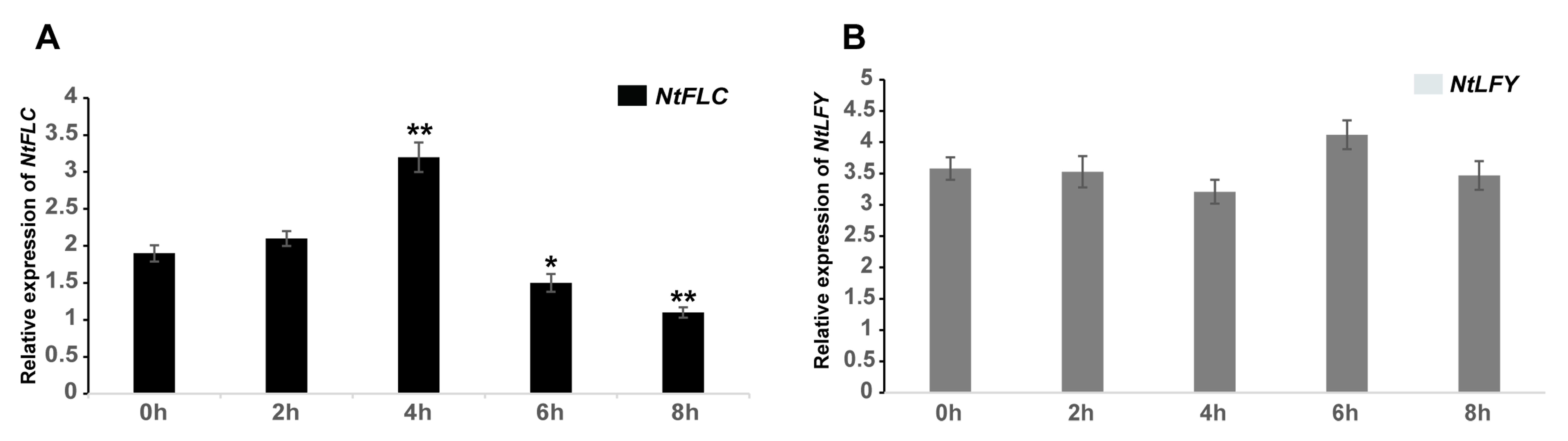

Supplement: Supplementary Figure 5 — qRT-PCR of NtFLC and NtLFY genes in responding to BR treatment. The seedlings of HD plants were grow in the greenhouse (28°C, 16h light/8h dark). When sixth true leaf appeared, plants were treated with brassinolide, and samples were collected in 0h, 2h, 4h, 6h and 8h after cold treatment for qRT-PCR. Data are shown as the mean ± SD from three independent experiment replicates. Significant changes measured by Student’s t test (* means p < 0.05 and ** means p < 0.01). [file Image_5.jpeg]

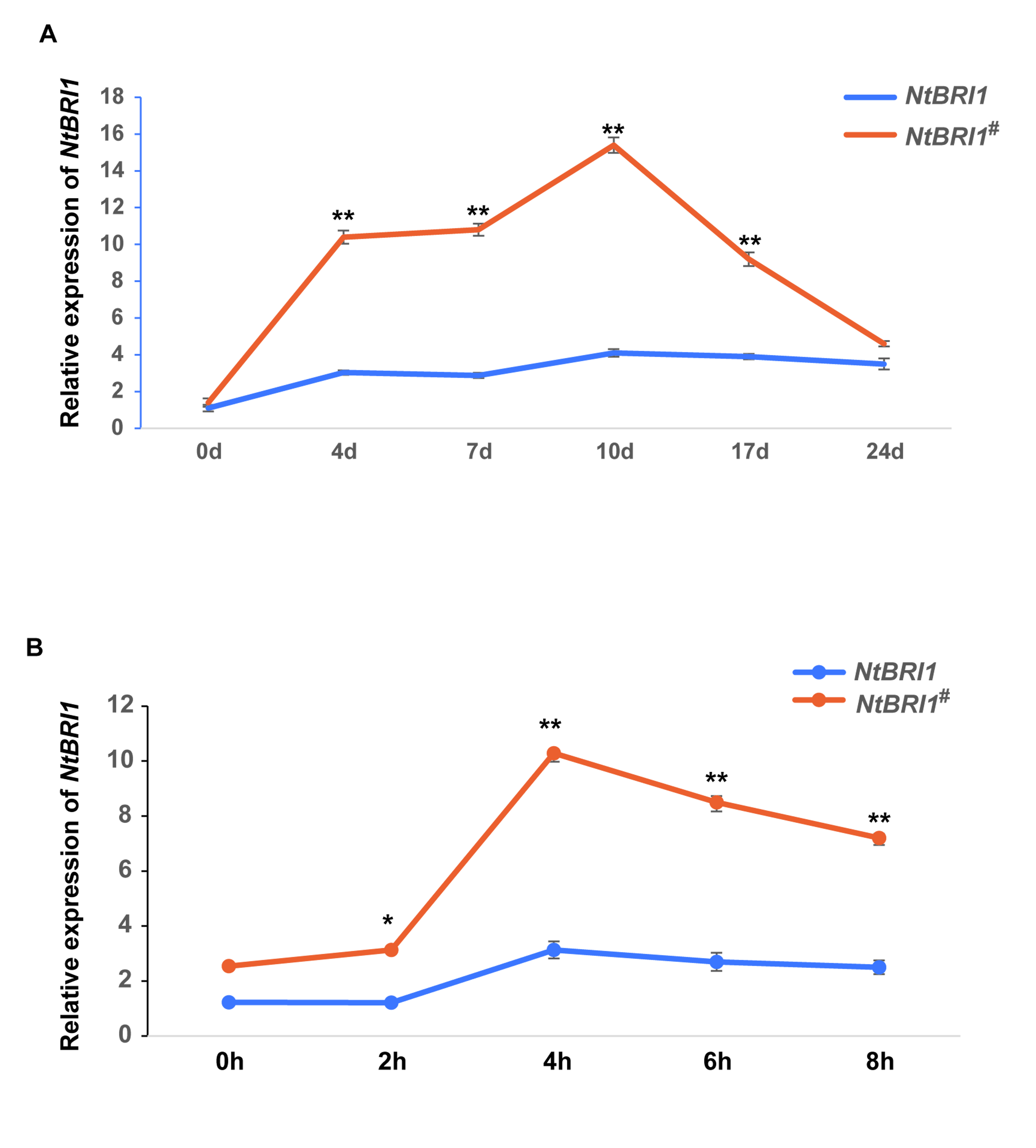

Supplement: Supplementary Figure 6 — qRT-PCR of NtBRI1 in responding to BR treatment and cold stress. For BR treatment, the seedlings of HD plants were grow in the greenhouse (28°C, 16h light/8h dark). When sixth true leaf appeared, plants were treated with brassinolide, and samples were collected in 0h, 2h, 4h, 6h and 8h after cold treatment for qRT-PCR. For cold stress treatment, the seedlings of HD plants were treated with cold stress (12°C for 10 days, 16h light/8h dark), and then all plants were moved into the greenhouse (28°C, 16h light/8h dark). The samples were collected in 1d, 4d, 7d, 10d, 17d and 24d after cold treatment for qRT-PCR (# means cold treatment). Data are shown as the mean ± SD from three independent experiment replicates. Significant changes measured by Student’s t test (* means p < 0.05 and ** means p < 0.01) [file Image_6.jpeg]
