# Supplementary material for: Transcriptome and functional analysis revealed the intervention of brassinosteroid in regulation of cold induced early flowering in tobacco
Source: Front Plant Sci. 2023 Mar 31;14:1136884. doi: 10.3389/fpls.2023.1136884 (PMC10102362; doi:10.3389/fpls.2023.1136884)
Supplement: Supplementary file 7 [file Table_1.docx]

Supplemental table 1 The primer sequences for gene cloning and qRT-PCR

|  | Names | Sequence 5'-3' | |
| --- | --- | --- | --- |
| Gene cloning | *NtBRI1-F* | GGATCCATGAAACCTCACAACAGTGCTA | |
|  | *NtBRI1-R* | ACTAGTTCATAGGTGTTTGCTCAGCTCAT | |
| Gene editing | *NtBRI1-Cas9-F* | GATTAGTAAGTTGGAGAAAGACTC | |
|  | *NtBRI1-Cas9-R* | AAACGAGTCTTTCTCCAACTTACT | |
| qRT-PCR | *NtFLC-F* | CTCAAGAAAATAGCAGCCTTCC | |
|  | *NtFLC-R* | TCTCCTTATTGCTCCTCACACA |  |
|  | *NtFLY-F* | GAACTCCTCGTCGGAGAAAG |  |
|  | *NtFLY-R* | ACTGGTTCCTCAGACAACCC |  |
|  | *NtBRI1-F* | GGCGATGGGTTTGTTGTTCT |  |
|  | *NtBRI1-R* | CTTCTCAAATGCGGCAAGGT |  |
|  | *β-actin-F* | AACAGTTTGGTTGGAGTTCTGG |  |
|  | *β-actin-R* | CATGAAGATTAAAGGCGGAGTG |  |
